# Development and validation of a machine learning model for evidence of prior or current HCV infection: a demographic screening approach for the US population

**DOI:** 10.1186/s12889-026-28311-2

**Published:** 2026-07-01

**Authors:** Dorian G Ding, Taoyi Chen, Yu Sheng, Jeffrey S. H. Lin, Ye Yuan

**Affiliations:** 1 College of Arts and Sciences, Emory University, Atlanta, USA; 2 School of Mathematics and Statistics, Nantong University, Nantong, China; 3 School of Biomedical Engineering, Tsinghua University, Beijing, China; 4 Department of Cellular and Physiological Sciences, Life Sciences Institute, University of British Columbia, Vancouver, Canada; 5 State Key Laboratory for Diagnosis and Treatment of Infectious Diseases, The First Affiliated Hospital, Zhejiang University School of Medicine, 15th Floor, Building 6, No. 79 Qingchun Road, Hangzhou, 310003 Zhejiang China

**Keywords:** Hepatitis C, Prevalence, Demographics, Screening, Machine learning

## Abstract

**Background:**

Hepatitis C virus (HCV) remains underdiagnosed in the United States despite recommendations for universal screening. A simple approach based on readily available demographic information may help target screening in settings where screening implementation continues to be incomplete.

**Methods:**

We analyzed 10 NHANES cycles (1999–2014 and 2017–2023) and defined HCV exposure as a positive HCV antibody or RNA result. Using sex, birth year, race/ethnicity, birthplace, and income-to-poverty ratio, we trained and compared logistic regression (LR) and machine learning models in training and cycle-based internal validation cohorts (48,434 and 20,762 participants, respectively). Model performance was evaluated based on sensitivity, specificity, positive predictive value (PPV), negative predictive value (NPV), and the area under the receiver operating characteristic curve (AUROC). A web-based calculator was developed to estimate HCV-marker positivity risk and support prioritization of confirmatory HCV testing.

**Results:**

69,196 participants were included, with 967 showing evidence of HCV exposure. Weighted HCV prevalence remained relatively stable across cycles, ranging from 1.22% to 1.93%. The prevalence did not change significantly after the pandemic. Earlier birth year, male sex, non-Hispanic Black race, US-born status, and lower income-to-poverty ratio were independently associated with HCV exposure. XGBoost performed better than LR in the validation cohort (AUROC 0.859 vs. 0.762, *p* < 0.001), but PPV remained low (5.1%). In the RNA-positive sensitivity analysis, XGBoost again showed the best validation performance (AUROC 0.867). Predicted risk showed clear risk stratification: observed HCV prevalence increased from 0.05% in the lowest-risk decile to 7.80% in the highest, with the top decile containing 57.2% of participants with HCV exposure and the top three deciles containing 86.2%.

**Conclusions:**

Five demographic variables were sufficient to build a supportive HCV-exposure risk stratification model in a nationally representative US sample. Most HCV-exposed individuals were concentrated in the highest predicted-risk groups, suggesting that this approach could help prioritize outreach and confirmatory testing where universal screening uptake remains incomplete. Because no laboratory data are required, it may also be practical in data-limited settings.

**Supplementary Information:**

The online version contains supplementary material available at https://doi.org/10.1186/s12889-026-28311-2.

## Introduction

Hepatitis C virus (HCV) remains a significant public health concern worldwide. In 2020, an estimated 57.8 million people were living with chronic HCV infection, and in 2022, there were 1 million newly infected individuals and 242,000 HCV-related deaths reported [1, 2]. In the United States, it was estimated that roughly 2.2 million adults were previously or currently infected with HCV [3]. Following acute infection, approximately 70% of HCV-infected individuals progress to chronic infection [2], and among them, about one-third of the patients will progress to cirrhosis, and the risk of liver-related mortality is 12-fold higher than the mortality of all other causes [4, 5]. HCV is composed of six major genotypes, of which genotypes 1 and 3 accounted for approximately 46% and 36% of global infections, respectively [6]. Although the development of direct-acting anti-viral (DAA) treatment has made virologic cure achievable across genotypes, with phase 3 sustained virologic response rates exceeding 95%, HCV remains a substantial source of morbidity and mortality worldwide [7].

Accordingly, the WHO set the elimination of viral hepatitis globally by 2030 as a major international goal, with HCV being one of its primary focuses [2]. The estimated number of HCV infections worldwide declined from 63.6 million in 2015 to 56.8 million in 2020, an 11% reduction; however, the WHO elimination target cannot be achieved at this pace [8]. Progress toward HCV elimination has been uneven across regions. In metropolitan Italy, HCV prevalence decreased, and awareness of HCV infection increased [9]. In sub-Saharan Africa, which accounts for approximately 11 million of the global infections, elimination efforts remained mild due to limited health infrastructure, low diagnostic coverage, and insufficient treatment access [8, 10]. Even in the US, underdiagnosis remains a central problem, with about 30% to 40% of US adults with past or present infection being unaware of their HCV-infected status [11, 12]. Thus, identifying high-risk groups in the general population can help with the case-finding process in a cost-effective way and further contribute to achieving the 2030 target.

Historically, HCV detection relied on risk-based testing, which targeted individuals with recognized exposure or conditions, including people who inject drugs, men who have sex with men (MSM), recipients of blood transfusions or organ transplants, and patients undergoing hemodialysis, before universal adult screening was recommended [13–15]. However, the real-world implementation of the screening recommendations has remained incomplete. Only 40.6% of pregnant individuals were screened in 2021, despite being a clearly recommended population for testing [16]. Similarly, strengthening screening among incarcerated populations is necessary, since their HCV positivity rates are about ten times higher than the general population [17]. Thus, a simple, low-cost, and easily replicable approach for early HCV risk stratification is needed to complement the universal screening recommendation.

Recent advancements in data science have expanded the use of machine learning methods such as random forest in clinical prediction due to their ability to capture the complex nonlinear relationships between variables, where classical statistical methods such as logistic regression (LR) often act as a benchmark [18, 19]. In structured tabular datasets, which are typical for electronic health records, tree-based machine learning methods were shown to outperform deep learning in medium-sized (~ 10k) datasets under most scenarios [20]. In a previous HCV risk prediction study, the best-performing machine-learning model was trained on 238 different predictors, with a C statistic of 0.916 [21]. Because the model relied on a large number of variables, its direct application may be less practical. Therefore, we developed, validated, and compared the performance of five different machine learning models and LR that were trained on only five demographic variables, using NHANES as a proof-of-concept dataset to demonstrate a framework that could be applied in resource-limited settings.

## Methods

### Study design and population

The National Health and Nutrition Examination Survey (NHANES) is a nationally representative, repeated cross-sectional survey of the US population that collects questionnaire data, examination measures, and laboratory results, and uses sampling weights to generate population-level estimates. The newly released 2021–2023 cycle makes it possible to extend prior HCV studies into the post-pandemic period [3]. This study used data from ten cycles (1999–2014, 2017–2023) from the NHANES database, with cycle 2015–2016 excluded because HCV-related laboratory data were not available. The protocol of NHANES was approved by the National Center for Health Statistics, and all participants in the database provided written informed consent [22]. 109,584 distinct participants were identified from 1999 to 2000 through 2021–2023. 31,863 participants were excluded due to unresolved HCV status (missing both HCV antibody status and RNA status), and 8,525 participants were excluded due to missing demographic data (birth year, sex, race/ethnicity, birthplace, or income-to-poverty ratio). These exclusion criteria resulted in a final modeling cohort of 69,196 participants (Supplementary Fig. 1).

### Outcome definition

The primary outcome of this study was HCV exposure, defined as either a positive HCV antibody test or a positive HCV RNA test. This endpoint was selected because HCV antibody or RNA positivity identifies individuals with evidence of prior or current HCV markers who would warrant guideline-based confirmatory evaluation, including RNA testing when needed. Participants were classified as HCV-negative if all available HCV-related marker(s) were negative. Participants were classified as having current infection if they tested positive for HCV RNA, regardless of antibody status. Participants were defined as having resolved HCV infection if they were positive for the antibody test but negative for the RNA test.

### Assessment of covariates

Covariates for baseline characterization were derived from official NHANES variables and standardized across cycles. Sex was recorded as male or female, and race/ethnicity was grouped as White, Black, Hispanic, and Asian/Other. Birthplace was dichotomized to US-born versus non-US-born. The income-to-poverty ratio (IPR) was defined as the ratio of family income to the federal poverty threshold. Education was categorized as less than college versus college or above, and marital status as married/living with partner versus other from NHANES questionnaire responses. Diabetes Mellitus (DM) and Coronary Artery Disease (CAD) were defined by self-reported physician diagnosis. Current smoking was defined as having smoked at least 100 cigarettes in life and currently smoking every day or some days. Alcohol use was classified as > 1 versus ≤ 1 drink on drinking days in the past 12 months. Probable depression was defined as a Patient Health Questionnaire-9 (PHQ-9) total score of ≥ 10 [23].

### Model training and validation

The study cohort was divided into a training cohort and an internal validation cohort in a 7:3 ratio according to the cycle information [24]. The training cohort included NHANES participants from cycles 1999–2000, 2003–2004, 2007–2008, 2009–2010, 2011–2012, 2013–2014, and 2017-March 2020 pre-pandemic, with a sample size of 48,434 [25]. The validation cohort included NHANES participants from the cycles 2001–2002, 2005–2006, and 2021–2023, with a population of 20,762. This split was selected to preserve an approximately 7:3 training-validation ratio while including both earlier and more recent NHANES cycles in the model-development and internal-validation cohorts. Five demographic and socioeconomic variables, including sex, birth year, race/ethnicity, birthplace, and income-to-poverty ratio, were used to train machine learning models to predict the HCV exposure status of the individuals. These variables were selected a priori because birth year, birthplace, sex, race/ethnicity, and poverty-related measures had been associated with HCV prevalence in the US in previous studies [11, 12, 26, 27]. Six models were trained, validated, and compared on the training and validation cohorts using the selected features. The models included LR, weighted logistic regression (WLR), random forest (RF), extreme gradient boosting (XGB), generalized additive model (GAM), and elastic-net logistic regression (GLMNET). The LR was chosen as an interpretable benchmark. RF and XGB were decision-tree-based models, whereas GAM modeled nonlinear associations using smooth functions; WLR and GLMNET were regression-based models. Both tree-based models and regularized logistic regression methods were well-suited to structured tabular health data and can capture nonlinear associations [18, 19, 28]. For XGBoost, boosting rounds were selected using 5-fold cross-validation within the training cohort only, with early stopping after 30 rounds without improvement. The validation cohort was not used for hyperparameter tuning or early stopping. Technical details of these models can be found in Supplementary Table 1. Predicted probabilities were calibrated using Platt scaling fit on the training cohort, and calibrated probabilities were used for decile-based display.

### Web-based calculator

To facilitate the practical use of the results of this study, the best-performing model was deployed as a web-based HCV-risk calculator, available at https://sylviesaiko.github.io/hcv-risk-calculator/. The tool provided an easy-to-use user interface in which the HCV exposure risk was calculated from the five demographic variables entered by healthcare personnel (Supplementary Fig. 2). It is intended to support outreach and confirmatory testing prioritization where universal screening is incompletely implemented, and should not be used to diagnose current infection or determine treatment eligibility without HCV RNA testing. The web calculator applies the same Platt-scaling calibration fitted in the fixed-cycle training cohort for probability display [29–31]. To support transparency and reproducibility, the calculator code is version-controlled in a public GitHub repository (https://github.com/SylvieSaiko/hcv-risk-calculator). The browser-readable model object used for scoring is provided in model-data.js, with additional exported model artifacts available as JSON files in the repository. Scoring is performed client-side in the browser after the page is opened.

### Statistical analysis

The weighted analyses adhered to NHANES analytic guidelines, which accounted for survey strata, primary sampling units, and mobile examination center (MEC) weights, and the resultant sample is representative of a large portion of the US population. Model training and primary machine-learning performance estimates were unweighted, except for weighted logistic regression, because most machine-learning algorithms used here do not directly incorporate the full NHANES complex survey design. As a sensitivity analysis, we calculated survey-weighted validation AUROC using pooled MEC weights. Model performance metrics included sensitivity, specificity, positive predictive value (PPV), negative predictive value (NPV), and the area under the receiver operating characteristic curve (AUROC), Brier score, calibration slope, calibration intercepts, and calibration plots. To assess potential subgroup differences, we evaluated the best-performing model validation performance by sex, race/ethnicity, birthplace, birth cohort, and income-to-poverty ratio. Subgroup analyses included AUROC, sensitivity, false-negative rate, specificity, PPV, Brier score, calibration intercept, and calibration slope. We also performed decision-curve analysis comparing XGBoost, standard LR, screen-all, and screen-none strategies across plausible calibrated-risk thresholds. Threshold-dependent performance metrics were calculated using a model-specific cutoff, defined as the 80th percentile of predicted risk within the corresponding dataset, such that the top 20% of participants were classified as high risk. Because no implementation threshold was specified a priori, we performed exploratory threshold analyses across Platt-calibrated XGBoost risk thresholds from 1.5% to 5.0%. p-values were unweighted for model-development contexts (train, validation, or modeling cohort compatibility), while survey-weighted p-values were used for the pre/post prevalence and weighted regression. The Pearson chi-square test was used for all categorical variables, and the Wilcoxon rank-sum test was used for all continuous variables in baseline comparisons. The DeLong test was used to generate p-values for AUROC comparison between different machine-learning models and LR. Model interpretability was assessed using Shapley Additive Explanations (SHAP) in the validation cohort. For each fitted model, the SHAP value was computed on a random validation subsample (up to 400 participants) using Monte Carlo estimation. The feature importance was summarized as the mean absolute SHAP value, and both global-level and participant-level SHAP distributions were visualized. Because birth year was the dominant predictor in SHAP analysis, we also performed a birth-year-only sensitivity analysis in which models were trained using birth year as the sole predictor and compared with the full five-variable models in the validation cohort. As a sensitivity analysis, model training and validation were repeated for all models after redefining the outcome as current HCV infection. Participants with negative HCV antibody results or those with positive antibody but negative RNA results were classified as controls, whereas antibody-positive participants without available RNA results were excluded from the analysis. The same five predictors were used, and the participants were split into training and validation cohorts in the same way as the main analysis. All analyses were performed using R (version 4.5.2; http://www.R-project.org).

## Results

### Prevalence of HCV in the United States

**Fig. 1:**
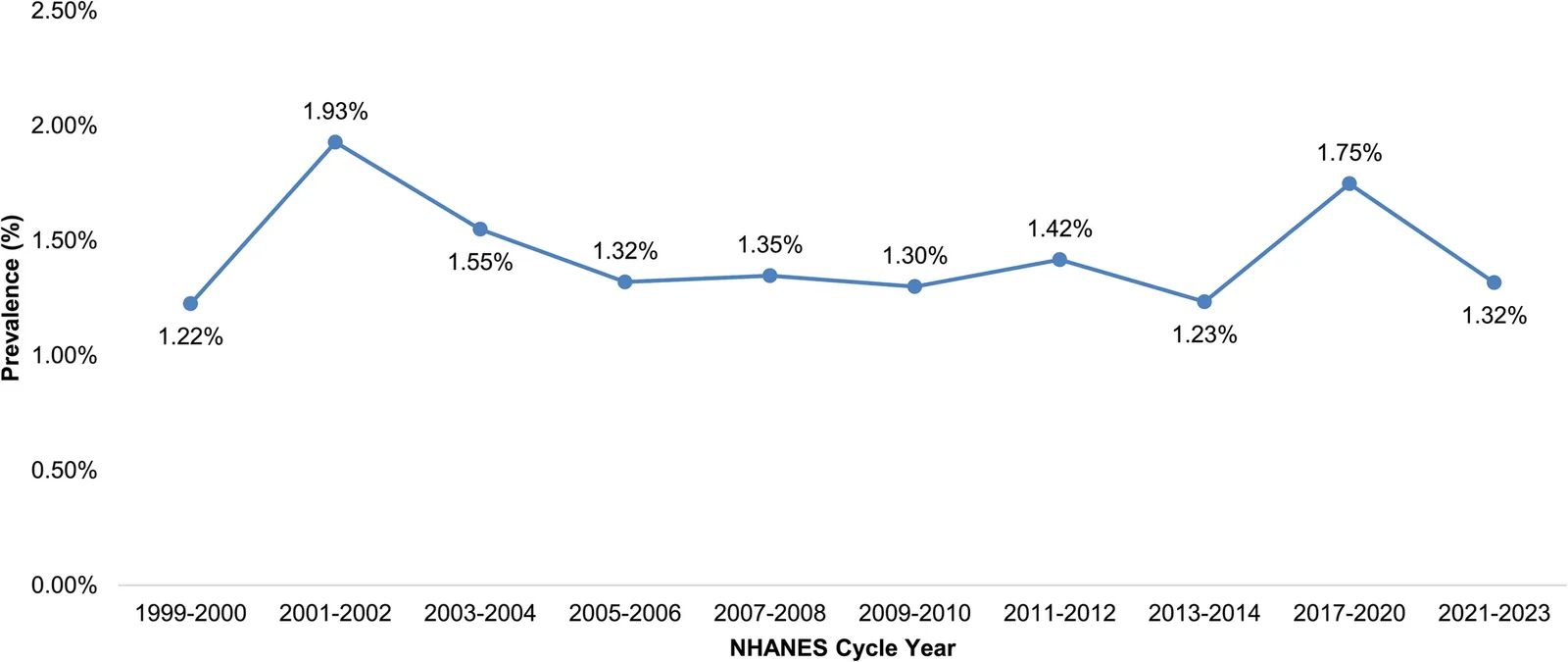
Weighted HCV prevalence by NHANES cycle year

A total of 69,196 participants were included in the analysis, with 967 individuals showing evidence of HCV exposure (Supplementary Fig. 1). The weighted HCV prevalence remained relatively stable across most cycles, ranging from 1.22% to 1.93%. The weighted prevalence did not differ significantly before and after the pandemic, with estimates of 1.71% (95% CI 1.08%-2.69%) in 2017–2020 and 1.38% (95% CI 0.98%-1.95%) in 2021–2023 **(**Fig. 1**)**. The HCV prevalence was higher in men than in women consistently over cycles (Supplementary Fig. 3a). The HCV prevalence was generally the highest in non-Hispanic Black participants, although non-Hispanic White prevalence also increased in the 2017–2020 pre-pandemic cycle (Supplementary Fig. 3b).

### Baseline characteristics of the analytical sample

The overall cohort was 48.84% male, 42.23% non-Hispanic White, 22.47% non-Hispanic Black, 26.77% Hispanic, 8.53% Asian or other, and 82.01% US-born **(**Table 1**)**. Compared with participants without HCV exposure, HCV-exposed participants were more likely to be male, older, Black, US-born, have diabetes, or have coronary artery disease. Participants with HCV exposure also had higher Hemoglobin A1C, glucose levels, alanine aminotransferase (ALT), aspartate aminotransferase (AST), gamma-glutamyl transferase (GGT), and creatinine. They were also more socioeconomically disadvantaged, with a lower income-to-poverty ratio, a lower level of education, lower insurance coverage, and higher rates of current smoking and probable depression. LDL was slightly lower rather than higher in the HCV-exposed group, and alkaline phosphatase did not differ significantly.

**Table 1:** Baseline characteristics by HCV exposure / No HCV exposure

Variable	Overall (*n* = 69,196)	HCV exposure (*n* = 967)	No HCV exposure (*n* = 68,229)	*p* value
Male sex	48.84	62.15	48.65	< 0.001
Birth year	1976 (1955–1991)	1958 (1952–1964)	1976 (1955–1991)	< 0.001
1911–1930	4.57	2.28	4.60	
1931–1950	16.37	18.92	16.34	
1951–1970	23.82	67.53	23.18	
1971–1990	30.16	10.13	30.45	
1991–2010	25.08	1.14	25.43	
Race/ethnicity				< 0.001
Non-Hispanic White	42.23	41.26	42.24	
Non-Hispanic Black	22.47	34.85	22.30	
Hispanic	26.77	17.79	26.90	
Asian/other	8.53	6.10	8.56	
Birth place: USA-born	82.01	92.04	81.87	< 0.001
Education (*n* = 54,663)				< 0.001
Less than college	79.64	93.24	79.40	
College or above	20.36	6.76	20.60	
Health insurance (*n* = 48,516)				< 0.001
Yes	83.15	75.07	83.27	
No	16.85	24.93	16.73	
Marital status (*n* = 51,622)				< 0.001
Married or living with partner	53.61	47.38	53.73	
Widowed, divorced, separated, or never married	46.39	52.62	46.27	
Alcohol use in past 12 months (*n* = 29,693)				< 0.001
>1/day	65.12	82.50	64.77	
<=1/day	34.88	17.50	35.23	
Smoking (*n* = 35,220)				< 0.001
Every day or some days	27.02	73.74	26.08	
Never	72.98	26.26	73.92	
Depression (*n* = 32,990)				< 0.001
Probable case	9.35	20.65	9.12	
Not a probable case	90.65	79.35	90.88	
Diabetes (*n* = 68,046)	8.33	15.96	8.23	< 0.001
CAD (*n* = 46,223)	4.41	6.58	4.36	< 0.001
Income to poverty ratio (*n* = 69,196)	2.0 (1.0-3.9)	1.3 (0.8–2.4)	2.0 (1.0-3.9)	< 0.001
BMI, kg/m2 (*n* = 68,202)	26.0 (21.6–30.8)	27.1 (23.5–31.1)	25.9 (21.6–30.8)	< 0.001
HbA1c, % (*n* = 60,318)	5.4 (5.1–5.7)	5.5 (5.2–5.9)	5.4 (5.1–5.7)	< 0.001
Glucose, mg/dl (*n* = 29,644)	97.1 (90.8-106.4)	102.5 (94.0-113.0)	97.0 (90.7–106.0)	< 0.001
HDL, mg/dl (*n* = 55,039)	51.0 (43.0–62.0)	50.0 (41.0–62.0)	51.0 (43.0–62.0)	0.039
LDL, mg/dl (*n* = 29,569)	104.0 (83.0-130.0)	98.0 (80.0-126.0)	104.0 (83.0-130.0)	0.004
TG, mg/dl (*n* = 30,485)	96.0 (66.0-144.0)	98.5 (71.0-145.2)	96.0 (66.0-144.0)	0.136
ALT, U/L (*n* = 59,613)	19.0 (15.0–26.0)	33.0 (21.0–55.0)	19.0 (15.0–26.0)	< 0.001
AST, U/L (*n* = 59,568)	22.0 (18.0–26.0)	33.0 (23.0–51.0)	22.0 (18.0–26.0)	< 0.001
ALP, U/L (*n* = 59,700)	74.0 (59.0–95.0)	75.0 (61.0–95.0)	74.0 (59.0–95.0)	0.349
GGT, U/L (*n* = 59,699)	18.0 (13.0–28.0)	39.0 (22.0–86.0)	18.0 (13.0–27.0)	< 0.001
TBil, mg/dl (*n* = 53,233)	0.6 (0.4–0.8)	0.6 (0.4–0.8)	0.6 (0.4–0.8)	0.041
Creatinine, mg/dl (*n* = 59,704)	0.8 (0.7-1.0)	0.9 (0.7-1.0)	0.8 (0.7-1.0)	< 0.001
Platelets, x10^9/L (*n* = 69,106)	258.0 (217.0-305.0)	230.5 (181.0-278.0)	258.0 (218.0-305.0)	< 0.001

Categorical variables are shown as percentages; continuous variables are shown as median (Q1-Q3). HCV exposure was defined as positivity for HCV antibody or HCV RNA. Variable-specific sample sizes are shown in the row labels because data were not available for all participants across all NHANES cycles. P values compare HCV-exposed and non-exposed participants

*HCV *hepatitis C virus, *CAD *coronary artery disease, *BMI *body mass index, *HbA1c *hemoglobin A1c, *HDL *high-density lipoprotein, *LDL *low-density lipoprotein, *TG *triglycerides, *ALT *alanine aminotransferase, *AST *aspartate aminotransferase, *ALP *alkaline phosphatase, *GGT *gamma-glutamyl transferase, *TBil *total bilirubin

### Characteristics of current vs. previous HCV infection

Current-versus-previous infection status could be determined for 888 participants who were HCV antibody positive and had available RNA results. Among these participants, 580 had current HCV infection, and 308 had previous HCV infection (Supplementary Table 2). Compared with previous infection, current HCV infection was more common in men and non-Hispanic Black participants and was associated with lower insurance coverage, lower income-to-poverty ratio, and higher smoking prevalence. Current infection also showed more adverse liver-related laboratory results, including higher alanine aminotransferase, aspartate aminotransferase, gamma-glutamyl transferase, and total bilirubin, together with lower platelet counts. Across cycles, the weighted proportion of current infection among antibody-positive participants declined from over 80% in the early cycles to 40.9% in 2017–2020 and 31.9% in 2021–2023.

### Key predictor identification

All six models were trained on five sociodemographic variables, including race/ethnicity, biological sex, birth year, birthplace, and income-to-poverty ratio. In a multivariable LR analysis **(**Table 2**)**, later birth year was associated with a lower chance of HCV exposure (adjusted OR per 10-year increase, 0.72, 95% CI 0.70–0.75, *p* < 0.001), whereas male sex (adjusted OR 1.91, 95% CI 1.63–2.23, *p* < 0.001), non-Hispanic Black race versus White (adjusted OR 1.87, 95% CI 1.56–2.24, *p* < 0.001), US-born status (adjusted OR 3.04, 95% CI 2.23–4.13, *p* < 0.001), and lower income-to-poverty ratio (adjusted OR per 1-unit increase, 0.69, 95% CI 0.65–0.73, *p* < 0.001) were independently associated with HCV exposure. Hispanic ethnicity (adjusted OR 1.08, 95% CI 0.85–1.38, *p* = 0.523) and Asian/Other race (adjusted OR 1.30, 95% CI 0.90–1.89, *p* = 0.162) were not independently associated with HCV exposure after adjustment.

**Table 2:** Unadjusted and adjusted logistic-regression predictors for HCV exposure

Term	Unadjusted OR	*p*-value	Adjusted OR	*p* value
Birth year (per 10 year)	0.76 (0.73, 0.78)	< 0.001	0.72 (0.70, 0.75)	< 0.001
Male sex	1.76 (1.51, 2.06)	< 0.001	1.91 (1.63, 2.23)	< 0.001
Black vs. White	1.59 (1.34, 1.89)	< 0.001	1.87 (1.56, 2.24)	< 0.001
Hispanic vs. White	0.64 (0.51, 0.79)	< 0.001	1.08 (0.85, 1.38)	0.523
Asian/Other vs. White	0.54 (0.38, 0.77)	< 0.001	1.30 (0.90, 1.89)	0.162
US-born vs. non-US-born	2.53 (1.93, 3.33)	< 0.001	3.04 (2.23, 4.13)	< 0.001
Income/poverty-line ratio (per 1 unit)	0.76 (0.72, 0.80)	< 0.001	0.69 (0.65, 0.73)	< 0.001

Odds ratios (ORs) and 95% CIs were estimated using logistic regression. The adjusted model included birth year, sex, race/ethnicity, birthplace, and income-to-poverty ratio. Birth year was modeled per 10-year increase, and income-to-poverty ratio was modeled per 1-unit increase. White race/ethnicity and non-US-born status were used as reference categories where applicable

### Model performance

**Fig. 2:**
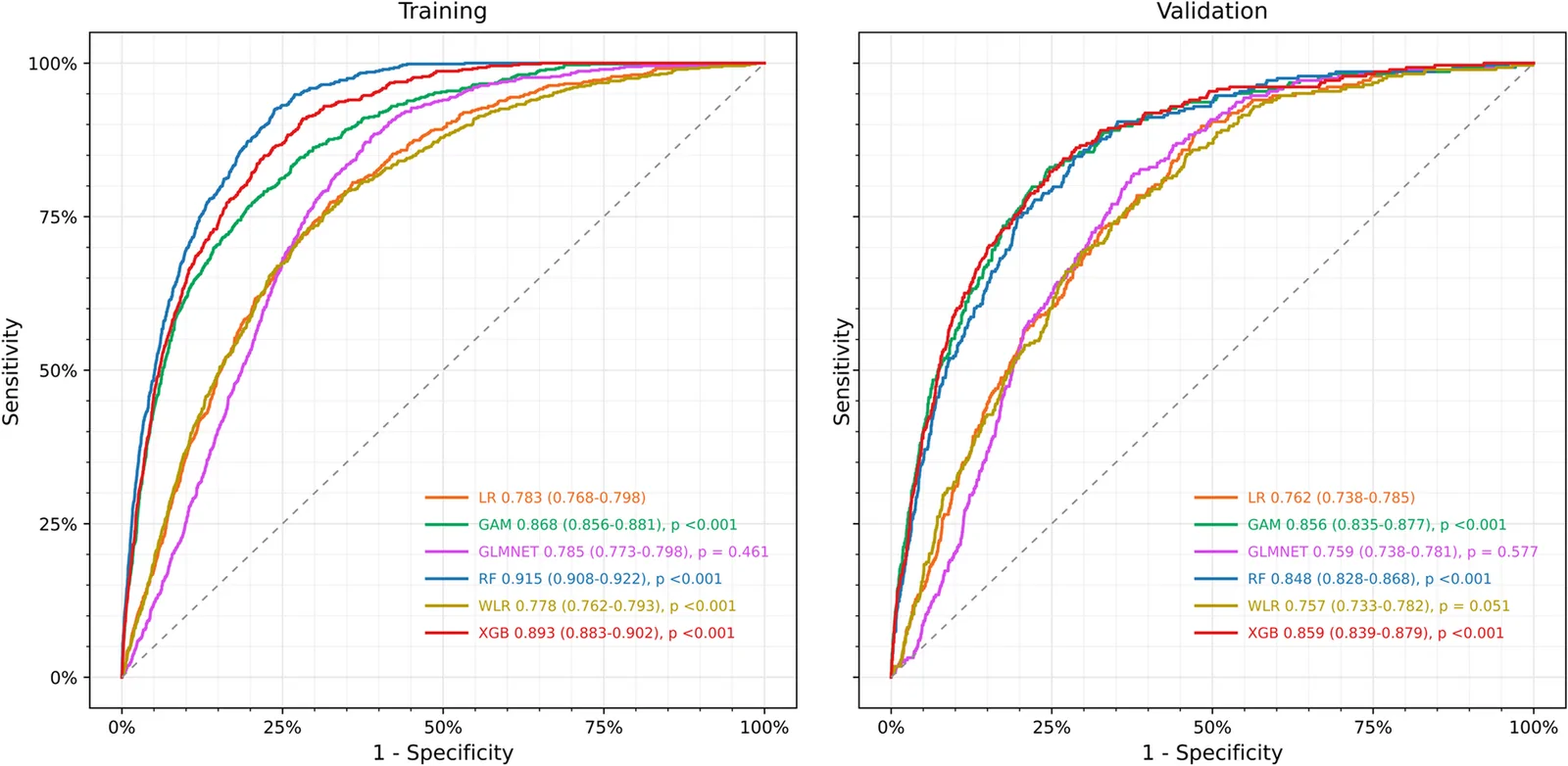
ROC curve comparison across models

The fixed-cycle split yielded 48,434 training participants and 20,762 validation participants (Supplementary Table 3). In the training set, RF achieved the highest apparent discrimination (AUROC 0.915), followed by XGBoost (AUROC 0.893), whereas LR achieved an AUROC of 0.783 (Table 3; Fig. 2). In the internal validation set, LR achieved an AUROC of 0.762 (95% CI, 0.738–0.785), sensitivity of 0.527 (95% CI, 0.468–0.584), specificity of 0.804 (95% CI, 0.799–0.810), PPV of 0.036 (95% CI, 0.031–0.042), and NPV of 0.992 (95% CI, 0.990–0.993). XGBoost achieved an AUROC of 0.859 (95% CI, 0.839–0.879), sensitivity of 74.9% (95% CI, 69.6%-79.6%), specificity of 80.8% (95% CI, 80.2%-81.3%), PPV of 5.1% (95% CI, 4.5%-5.8%), and NPV of 99.6% (95% CI, 99.5%-99.7%). Validation Brier scores were similar after Platt calibration, while XGBoost had higher PR-AUC than LR. (Table 3; Fig. 2).

**Table 3:** Model performance for HCV exposure prediction

Cohort	Model	Sensitivity	Specificity	PPV	NPV	AUROC	PR-AUC	Brier score	Calibration intercept	Calibration slope
Training	LR	0.583 (0.546–0.620)	0.805 (0.802–0.809)	0.041 (0.037–0.045)	0.993 (0.992–0.993)	0.783 (0.768–0.798)	0.041 (0.037–0.047)	0.014 (0.013–0.015)	0.000 (-0.076-0.076)	1.000 (0.921–1.079)
	GAM	0.757 (0.724–0.788)	0.808 (0.804–0.811)	0.053 (0.049–0.058)	0.996 (0.995–0.996)	0.868 (0.856–0.881)	0.103 (0.091–0.121)	0.013 (0.012–0.014)	0.000 (-0.077-0.077)	1.000 (0.933–1.067)
	GLMNET	0.522 (0.484–0.559)	0.805 (0.801–0.808)	0.037 (0.033–0.041)	0.992 (0.991–0.992)	0.785 (0.773–0.798)	0.034 (0.031–0.038)	0.014 (0.013–0.015)	0.000 (-0.076-0.076)	1.000 (0.917–1.083)
	RF*	0.864 (0.836–0.888)	0.810 (0.806–0.813)	0.061 (0.056–0.066)	0.998 (0.997–0.998)	0.915 (0.908–0.922)	0.141 (0.121–0.162)	0.013 (0.012–0.014)	0.000 (-0.079-0.079)	1.000 (0.941–1.059)
	WLR	0.577 (0.540–0.614)	0.805 (0.802–0.809)	0.041 (0.037–0.045)	0.993 (0.992–0.993)	0.778 (0.762–0.793)	0.041 (0.037–0.047)	0.014 (0.013–0.015)	0.000 (-0.076-0.076)	1.000 (0.919–1.081)
	XGB	0.801 (0.770–0.829)	0.808 (0.804–0.811)	0.056 (0.052–0.061)	0.996 (0.996–0.997)	0.893 (0.883–0.902)	0.112 (0.097–0.128)	0.013 (0.012–0.014)	0.000 (-0.078-0.078)	1.000 (0.936–1.064)
Validation	LR	0.527 (0.468–0.584)	0.804 (0.799–0.810)	0.036 (0.031–0.042)	0.992 (0.990–0.993)	0.762 (0.738–0.785)	0.035 (0.029–0.046)	0.013 (0.012–0.015)	0.021 (-0.098-0.139)	0.843 (0.729–0.957)
	GAM	0.753 (0.699–0.799)	0.808 (0.802–0.813)	0.051 (0.045–0.058)	0.996 (0.995–0.997)	0.856 (0.835–0.877)	0.096 (0.074–0.125)	0.013 (0.011–0.014)	0.064 (-0.056-0.184)	0.939 (0.839–1.038)
	GLMNET	0.519 (0.461–0.577)	0.804 (0.799–0.810)	0.035 (0.030–0.041)	0.992 (0.990–0.993)	0.759 (0.738–0.781)	0.030 (0.026–0.038)	0.013 (0.012–0.015)	-0.003 (-0.122-0.115)	0.825 (0.706–0.945)
	RF	0.731 (0.677–0.780)	0.807 (0.802–0.813)	0.050 (0.044–0.057)	0.995 (0.994–0.996)	0.848 (0.828–0.868)	0.079 (0.061–0.104)	0.013 (0.012–0.014)	0.062 (-0.060-0.184)	0.713 (0.640–0.786)
	WLR	0.519 (0.461–0.577)	0.804 (0.799–0.810)	0.035 (0.030–0.041)	0.992 (0.990–0.993)	0.757 (0.733–0.782)	0.036 (0.029–0.048)	0.013 (0.012–0.015)	0.029 (-0.090-0.147)	0.846 (0.731–0.962)
	XGB*	0.749 (0.696–0.796)	0.808 (0.802–0.813)	0.051 (0.045–0.058)	0.996 (0.995–0.997)	0.859 (0.839–0.879)	0.092 (0.072–0.115)	0.013 (0.012–0.014)	0.068 (-0.053-0.189)	0.855 (0.767–0.943)

Model performance was evaluated in the training and validation cohorts. Values are estimates with 95% confidence intervals. Sensitivity, specificity, positive predictive value (PPV), and negative predictive value (NPV) were calculated using the cohort-specific 80th-percentile threshold for each model for fixed-capacity comparison. Brier score, calibration intercept, and calibration slope were estimated using Platt-calibrated predicted risks. For XGBoost, boosting rounds were selected by 5-fold cross-validation within the training cohort only, with early stopping after 30 rounds without improvement

Asterisks indicate the best-performing model in each cohort according to AUROC

Calibration metrics are reported in Table 3, and calibration plots are shown in Supplementary Fig. 4. In the validation cohort, Platt-calibrated XGBoost and LR had similar Brier scores, while calibration intercepts were close to 0 and calibration slopes were below 1 for both models. The RF achieved the highest training AUROC (0.915) but lower validation performance (0.848), suggesting less stable generalization than XGBoost.

In survey-weighted validation sensitivity analyses, XGBoost AUROC decreased from 0.859 unweighted to 0.830 weighted, whereas LR AUROC was similar after weighting. XGBoost retained higher weighted discrimination than LR (Supplementary Table 4).

In the sensitivity analysis, where the outcome was changed to HCV RNA positivity, the overall pattern of model performance was similar (Supplementary Table 5, Supplementary Fig. 5). The fixed-cycle split resulted in 47,585 training participants and 20,688 validation participants, with 425 and 160 current HCV infections, respectively. In the training cohort, LR achieved an AUROC of 0.802, whereas RF was the best-performing model with an AUROC of 0.921. In the validation cohort, LR achieved an AUROC of 0.794, whereas XGBoost again showed the best performance with an AUROC of 0.867.

**Fig. 3:**
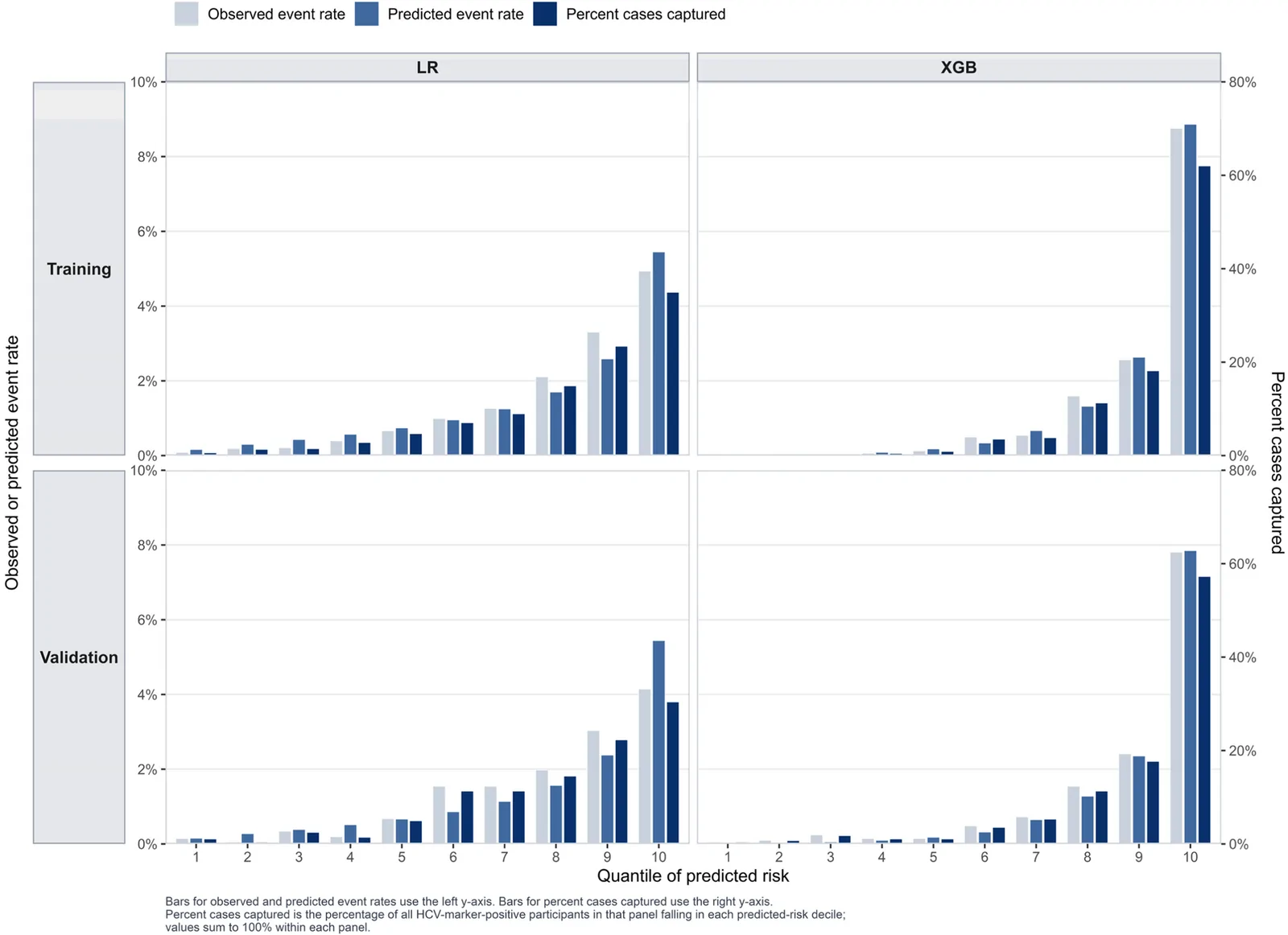
Observed and predicted HCV exposure prevalence and case capture by predicted-risk decile

**Fig. 4:**
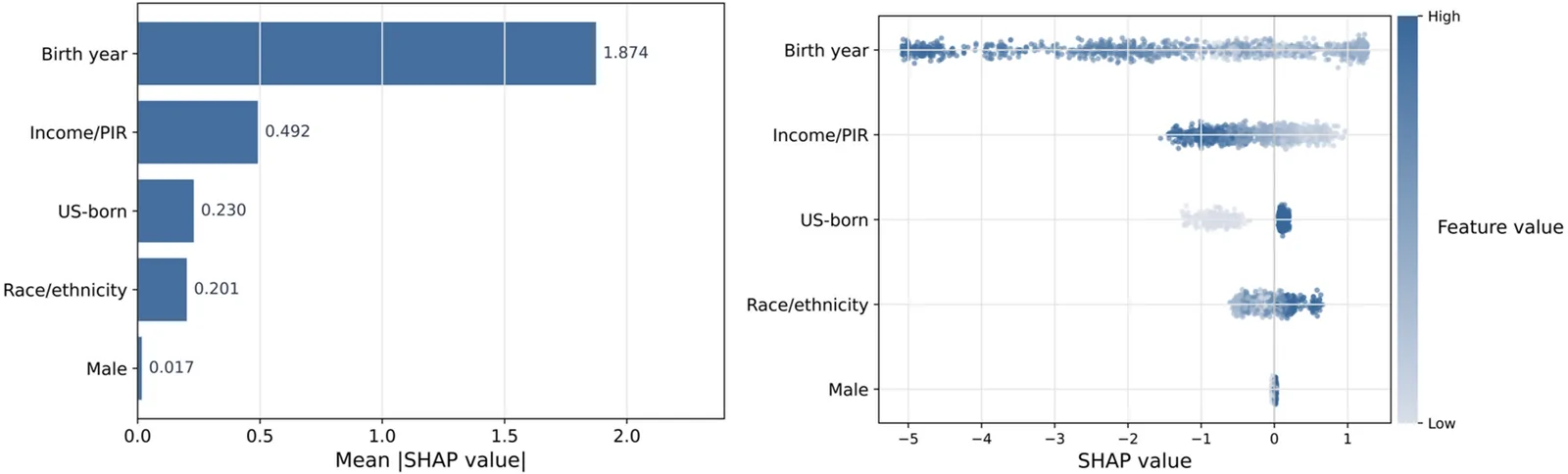
SHAP interpretation of the best-performing XGBoost model. **A** Mean absolute SHAP values. **B** Beeswarm plot

In the validation cohort, decile-based risk stratification showed a clear gradient in HCV exposure prevalence (Fig. 3, Supplementary Table 6). For the LR model, the observed prevalence increased from 0.14% in the lowest decile to 4.14% in the highest decile, suggesting a ~ 30-fold increase, whereas for XGBoost, the observed prevalence increased from 0.05% in the lowest decile to 7.80% in the highest decile, corresponding to an approximately 160-fold increase. The top validation decile captured 30.4% of participants with HCV exposure for LR and 57.2% for XGBoost. The top three deciles together captured approximately 86.2% of cases in XGBoost, compared to 67.1% in LR. Decision-curve analysis comparing XGBoost with standard LR showed higher net benefit for XGBoost across plausible low calibrated-risk thresholds (Supplementary Fig. 6). At calibrated-risk thresholds of 2% and 3%, XGBoost provided net-benefit gains of 3.3 and 3.9 per 1,000 participants compared with LR, respectively. In the XGBoost model, SHAP analysis identified birth year as the dominant predictor (mean |SHAP value| = 1.874), followed by income-to-poverty ratio (0.492), US-born status (0.230), race/ethnicity (0.201), and male sex (0.017) **(**Fig. 4A**)**. The birth-year-only sensitivity analysis showed that full XGBoost outperformed birth-year-only XGBoost in the validation cohort, with higher AUROC (0.859 vs. 0.790) and greater top-decile case capture (57.2% vs. 32.5%) (Supplementary Table 7). In subgroup analyses, XGBoost performance varied across demographic groups (Supplementary Table 8). Sensitivity was higher in males than females (83.5% vs. 61.9%; false-negative rate 16.5% vs. 38.1%) and was highest among Black participants (92.9%; false-negative rate 7.1%). Sensitivity was lower among Asian/Other participants (54.2%), Hispanic participants (61.5%), and non-US-born participants (23.8%), although some of these subgroups had relatively few HCV-marker-positive participants. By birth cohort, sensitivity was highest in the 1951–1970 cohort (91.4%) but lower in both older and younger cohorts. Calibration was generally acceptable in larger subgroups, although calibration estimates were imprecise in sparse subgroups with few HCV-marker-positive participants (Supplementary Table 8).

Because the 80th-percentile threshold was intended for fixed-capacity model comparison rather than deployment, we evaluated XGBoost performance across absolute Platt-calibrated risk thresholds (Supplementary Table 9). At a 2% calibrated-risk threshold, XGBoost classified 17.2% of validation participants as high risk and yielded sensitivity 71.4%, specificity 83.6%, PPV 5.7%, NPV 99.5%, and number needed to screen of 17.7. At a 3.5% threshold, XGBoost classified 10.0% as high risk, with sensitivity 57.2%, specificity 90.7%, PPV 7.8%, NPV 99.4%, and number needed to screen of 12.8. Decision-curve analysis showed higher net benefit for XGBoost than standard LR across these low calibrated-risk thresholds (Supplementary Fig. 6).

## Discussion

In this study, the prevalence of HCV exposure was stable across all cycles. There was no statistically significant difference between the 2017–2020 pre-pandemic period and the 2021–2023 cycle, suggesting that the pandemic did not have a significant impact on national HCV burden [11]. The prevalence was consistent with previous reports of HCV prevalence of approximately 1% [3]. Notably, the proportion of current infection among affected individuals dropped steadily, falling from above 80% in previous cycles to 31.9% in 2021–2023. This trend likely reflects the synergy between the expanded use of DAA treatment in 2014 and ongoing prevention efforts [8, 32]. HCV exposure prevalence was consistently higher in males than in females, and non-Hispanic Black participants had the highest weighted prevalence across all cycles except for 2017–2020. The prevalence shift was probably explained by the opioid epidemic, during which the new HCV infections surged among young white adults [33].

Evidence of HCV exposure was more common among males, non-Hispanic Black participants, US-born participants, and participants with greater socioeconomic disadvantage, which is consistent with previous findings and thus expected [11, 12, 26, 27]. HCV-exposed individuals also had significantly higher rates of current smoking and probable depression, showing the association between HCV infection and psychiatric comorbidity, which was shown to be both a risk factor for infection and a barrier to treatment [34]. The liver-related laboratory profile was notably more adverse in the HCV-exposed groups than in the general population. ALT, AST, and GGT were higher, indicating ongoing hepatic inflammation. Creatinine was also higher in the HCV-exposed group, which may suggest HCV-associated glomerulonephritis [35]. Interestingly, LDL was slightly lower in the HCV-exposed group, consistent with prior evidence that showed how HCV disrupts lipid metabolism by utilizing lipoprotein pathways for cell entry and assembly [36]. In the cohort, current HCV infection was associated with a more adverse socioeconomic and liver-related laboratory profile than those with resolved infection, which may reflect differences in viral clearance, treatment uptake, and access to care.

A central finding of this study is that a model based on five readily available demographic variables can still provide moderate-to-good HCV risk stratification, particularly for outreach prioritization. This model is not intended to replace universal HCV screening, but to support prioritization when universal screening is incompletely implemented or when outreach capacity is limited. Potential use cases include population outreach, community screening programs, correctional or transitional-care settings, and health systems that need to identify individuals most likely to benefit from active screening reminders or linkage-to-testing efforts. Importantly, the primary model estimates risk of prior or current HCV-marker positivity rather than active infection; therefore, its practical use is to prioritize confirmatory HCV testing and linkage pathways, not to diagnose current infection. These intended-use constraints are important because the model includes race/ethnicity, birthplace, and income-to-poverty ratio. These variables should be interpreted as markers of population-level epidemiology and structural exposure patterns rather than biological susceptibility, and model use should avoid stigmatizing or deprioritizing individuals outside historically high-risk profiles. Therefore, the tool should be used only as an adjunct to universal screening, with local validation, recalibration, and subgroup monitoring before implementation.

XGBoost had the best validation performance and identified high-risk groups more effectively than LR, and the incremental value was most evident in rank-based case concentration, where XGBoost identified a larger proportion of HCV-marker-positive participants within the same highest-risk outreach strata and showed higher decision-curve net benefit across plausible low testing thresholds. Although XGBoost improved discrimination and case concentration, PPV remained low because HCV-marker positivity was uncommon. The observed AUROC improvement should therefore not be interpreted as clinical superiority by itself. Its potential clinical and public-health relevance is that, at the same fixed outreach capacity, XGBoost concentrated more HCV-marker-positive participants in the highest-risk strata than LR, increasing top-decile case capture and lowering the number needed to screen. Thus, the model is better understood as an outreach-prioritization tool rather than a diagnostic tool.

The subgroup analysis showed that model performance was not uniform across groups, with higher sensitivity among males, Black participants, US-born participants, and the 1951–1970 birth cohort, but lower sensitivity among non-US-born participants and some younger or older birth cohorts. False-negative rates also differed across subgroups, particularly among non-US-born participants and younger birth cohorts. Subgroup calibration was broadly acceptable in larger groups but less precise in sparse subgroups; therefore, implementation would require local recalibration and ongoing subgroup monitoring. These findings support the model’s use as an outreach-prioritization tool rather than a replacement for universal screening. They also indicate that any implementation should include local validation, recalibration, and subgroup monitoring to reduce the risk of inequitable case-finding. We further evaluated XGBoost across absolute Platt-calibrated risk thresholds. This analysis showed how the model could be adapted to different testing capacities: for example, a 2.0% threshold classified 17.2% of validation participants as high risk and captured 71.4% of HCV-marker-positive participants, whereas a more resource-constrained 3.5% threshold classified 10.0% as high risk and captured 57.2%. Together with the decision-curve analysis, these findings support the model’s potential use for outreach prioritization.

Most previous HCV prediction studies have relied on much more detailed clinical data. Jang et al. reported stronger discrimination with an electronic health record (EHR) based model that included hundreds of candidate predictors in a state-level dataset [21]. Beyond individual-level risk prediction, recent European experience has also shown that systematic, data-driven identification of untreated or overlooked HCV patients through laboratory, pharmacy, and electronic health-record data may improve linkage to care and support HCV elimination strategies [37]. Our goal, however, was not to maximize predictive performance at all costs, but to evaluate how far a simple demographic model could go in a nationally representative sample. In that context, NHANES offers an important advantage because it is nationally representative, and the required sociodemographic variables are widely available in both clinical and public health settings. The SHAP analyses also highlighted the importance of the birth-cohort effect in HCV epidemiology, with birth year ranking as the most influential predictor across all models, consistent with prior findings [12]. However, the birth-cohort-only sensitivity analysis shows that the XGB model performed better on five variables, suggesting additional predictive information from the other demographic and socioeconomic variables. The similar performance observed in the sensitivity analysis that used current HCV infection as the endpoint further suggests that these demographic predictors retain useful discriminatory value even when the outcome is restricted to clinically active infection.

This study has several strengths. It leveraged multiple NHANES cycles, including the newly released 2021–2023 cycle, which could be weighted to represent the US population. Additionally, all models were interpreted using SHAP, which improves the transparency and interpretability of the results. Moreover, the best-performing model was published online with open access, which offers a practical application opportunity in clinical settings. In addition, the main finding remained mostly unchanged in a sensitivity analysis where only HCV RNA-positive individuals were considered positive, indicating the robustness of the model. The study also has important limitations. NHANES is cross-sectional for HCV infection status, so incident infection and longitudinal transitions cannot be assessed. A further limitation is potential selection bias related to missing HCV status and complete-case inclusion. Furthermore, NHANES excludes several populations with high HCV burden, including incarcerated people, people experiencing homelessness, and some institutionalized populations. NHANES also does not provide a validation sample specifically designed for key HCV risk groups such as people who inject drugs, men who have sex with men (MSM), or patients receiving hemodialysis. Therefore, the true national burden is likely underestimated [3]. This limitation is particularly important for public-health interpretation because these excluded groups are also settings where targeted outreach, linkage-to-testing, and linkage-to-care strategies may be most needed. The models were internally validated only due to the lack of other nationally representative datasets and have not been externally validated in independent health systems or non-US populations. In addition, most machine-learning models were trained without incorporating the full NHANES complex survey design; weighted validation analyses were added as sensitivity analyses, but external validation and recalibration would still be needed before implementation. Therefore, the performance of the models should not be interpreted as evidence that the model can be directly transported to other countries or healthcare settings without local validation and recalibration. Finally, the models should be interpreted as complementary tools for case-finding rather than replacements for guideline-recommended universal screening.

In summary, this study demonstrates that a parsimonious set of five sociodemographic variables was sufficient to stratify HCV exposure risk in the US population. The same framework could potentially be adopted and recalibrated using local data in other US healthcare systems or non-US populations, contributing to the global progress toward the WHO goal. Importantly, this model aligns with the current universal recommendation, and a high predicted risk score provides an additional reason for prioritizing HCV testing in selected participants, particularly in resource-limited settings where universal screening has not yet been adopted.

## Supplementary information

Supplementary Material 1.

## Data Availability

The datasets analyzed in this study are publicly available from the National Health and Nutrition Examination Survey (NHANES) repository of the US Centers for Disease Control and Prevention at https://www.cdc.gov/nchs/nhanes/. The web-based calculator is publicly available at https://sylviesaiko.github.io/hcv-risk-calculator/. The calculator source code is available in a public GitHub repository at https://github.com/SylvieSaiko/hcv-risk-calculator.

## References

[1] Cui F, Blach S, Manzengo Mingiedi C, et al. Global reporting of progress towards elimination of hepatitis B and hepatitis C. Lancet Gastroenterol Hepatol. 2023;8(4):332–42. 10.1016/s2468-1253(22)00386-7. 36764320

[2] World Health Organization, Hepatitis C, World Health O. 2024. https://www.who.int/news-room/fact-sheets/detail/hepatitis-c. Accessed 2026 April 1.

[3] Hall EW, Bradley H, Barker LK, et al. Estimating hepatitis C prevalence in the United States, 2017–2020. Hepatology. 2025;81(2):625–36. 10.1097/hep.0000000000000927. 38739849 PMC11557732

[4] Mahajan R, Xing J, Liu SJ, et al. Mortality among persons in care with hepatitis C virus infection: the Chronic Hepatitis Cohort Study (CHeCS), 2006–2010. Clin Infect Dis. 2014;58(8):1055–61. 10.1093/cid/ciu077. 24523214

[5] Gordon SC, Lamerato LE, Rupp LB, et al. Prevalence of cirrhosis in hepatitis C patients in the Chronic Hepatitis Cohort Study (CHeCS): a retrospective and prospective observational Study. Am J Gastroenterol. 2015;110(8):1169–77. 10.1038/ajg.2015.203. 26215529 PMC5731242

[6] Messina JP, Humphreys I, Flaxman A, et al. Global distribution and prevalence of hepatitis C virus genotypes. Hepatology. 2015;61(1):77–87. 10.1002/hep.27259. 25069599 PMC4303918

[7] Feld JJ, Jacobson IM, Hézode C, et al. Sofosbuvir and Velpatasvir for HCV Genotype 1, 2, 4, 5, and 6 Infection. N Engl J Med. 2015;373(27):2599–607. 10.1056/NEJMoa1512610. 26571066

[8] Polaris Observatory HCV, Collaborators. Global change in hepatitis C virus prevalence and cascade of care between 2015 and 2020: a modelling study. Lancet Gastroenterol Hepatol. 2022;7(5):396–415. 10.1016/s2468-1253(21)00472-6. 35180382

[9] Andriulli A, Stroffolini T, Mariano A, et al. Declining prevalence and increasing awareness of HCV infection in Italy: A population-based survey in five metropolitan areas. Eur J Intern Med. 2018;53:79–84. 10.1016/j.ejim.2018.02.015. 29475770

[10] Sonderup MW, Afihene M, Ally R, et al. Hepatitis C in sub-Saharan Africa: the current status and recommendations for achieving elimination by 2030. Lancet Gastroenterol Hepatol. 2017;2(12):910–9. 10.1016/S2468-1253(17)30249-2. 29132760

[11] Lewis KC, Barker LK, Jiles RB, Gupta N. Estimated Prevalence and Awareness of Hepatitis C Virus Infection Among US Adults: National Health and Nutrition Examination Survey, January 2017-March 2020. Clin Infect Dis. 2023;77(10):1413–5. 10.1093/cid/ciad411. 37417196 PMC11000503

[12] Zou B, Yeo YH, Le MH, et al. Prevalence of Viremic Hepatitis C Virus Infection by Age, Race/Ethnicity, and Birthplace and Disease Awareness Among Viremic Persons in the United States, 1999–2016. J Infect Dis. 2020;221(3):408–18. 10.1093/infdis/jiz479. 31560391

[13] US Preventive Services Task Force, Owens DK, Davidson KW, et al. Screening for Hepatitis C Virus Infection in Adolescents and Adults: US Preventive Services Task Force Recommendation Statement. JAMA. 2020;323(10):970–5. 10.1001/jama.2020.1123. 32119076

[14] Schillie S, Wester C, Osborne M, Wesolowski L, Ryerson AB. CDC Recommendations for Hepatitis C Screening Among Adults - United States, 2020. MMWR Recomm Rep. 2020;69(2):1–17. 10.15585/mmwr.rr6902a1. Published 2020 Apr 10. 32271723 PMC7147910

[15] Jin F, Dore GJ, Matthews G, et al. Prevalence and incidence of hepatitis C virus infection in men who have sex with men: a systematic review and meta-analysis. Lancet Gastroenterol Hepatol. 2021;6(1):39–56. 10.1016/S2468-1253(20)30303-4. 33217341

[16] Kaufman HW, Osinubi A, Meyer WA 3rd, et al. Hepatitis C Virus Testing During Pregnancy After Universal Screening Recommendations. Obstet Gynecol. 2022;140(1):99–101. 10.1097/AOG.0000000000004822. 35849464 PMC9205300

[17] McNamara M, Furukawa N, Cartwright EJ, Advancing Hepatitis C. Elimination through Opt-Out Universal Screening and Treatment in Carceral Settings, United States. Emerg Infect Dis. 2024;30(13):S80–7. 10.3201/eid3013.230859. 38561831 PMC10986823

[18] Khosravi B, Weston AD, Nugen F, et al. Demystifying Statistics and Machine Learning in Analysis of Structured Tabular Data. J Arthroplasty. 2023;38(10):1943–7. 10.1016/j.arth.2023.08.045. 37598784 PMC10947212

[19] Goldstein BA, Navar AM, Pencina MJ, Ioannidis JP. Opportunities and challenges in developing risk prediction models with electronic health records data: a systematic review. J Am Med Inf Assoc. 2017;24(1):198–208. 10.1093/jamia/ocw042. PMC5201180 27189013

[20] Borisov V, Leemann T, Sebler K, Haug J, Pawelczyk M, Kasneci G. Deep Neural Networks and Tabular Data: A Survey. IEEE Trans Neural Netw Learn Syst. 2024;35(6):7499–519. 10.1109/TNNLS.2022.3229161. 37015381

[21] Jang SC, Lo-Ciganic WH, Hernandez-Con P, et al. Development and Validation of a Machine Learning-Based Screening Algorithm to Predict High-Risk Hepatitis C Infection. Open Forum Infect Dis. 2025;12(8):ofaf496. 10.1093/ofid/ofaf496. Published 2025 Aug 15. 40874186 PMC12378832

[22] National Center for Health Statistics, Centers for Disease Control and Prevention. NHANES Research Ethics Review Board Approval. National Health and Nutrition Examination Survey. 2024. https://wwwn.cdc.gov/nchs/nhanes/default.aspx. Accessed 2026 April 1.

[23] Kroenke K, Spitzer RL, Williams JB. The PHQ-9: validity of a brief depression severity measure. J Gen Intern Med. 2001;16(9):606–13. 10.1046/j.1525-1497.2001.016009606.x. 11556941 PMC1495268

[24] Lin L, Zhang L, Zhang J, Ding D. A Novel Depression Risk Prediction Model Using NHANES Data With Mendelian Randomization Validation. Brain Behav. 2025;15(7):e70674. 10.1002/brb3.70674. 40714919 PMC12409830

[25] Ramrakhiani NS, Chen VL, Le M, et al. Optimizing hepatitis B virus screening in the United States using a simple demographics-based model. Hepatology. 2022;75(2):430–7. 10.1002/hep.32142. 34496066

[26] Rosenberg ES, Rosenthal EM, Hall EW, et al. Prevalence of Hepatitis C Virus Infection in US States and the District of Columbia, 2013 to 2016. JAMA Netw Open. 2018;1(8):e186371. 10.1001/jamanetworkopen.2018.6371. Published 2018 Dec 7. 30646319 PMC6324373

[27] Hofmeister MG, Rosenthal EM, Barker LK, et al. Estimating Prevalence of Hepatitis C Virus Infection in the United States, 2013–2016. Hepatology. 2019;69(3):1020–31. 10.1002/hep.30297. 30398671 PMC6719781

[28] Dentamaro V, Giglio P, Impedovo D, Pirlo G, Ciano MD. An Interpretable Adaptive Multiscale Attention Deep Neural Network for Tabular Data. IEEE Trans Neural Netw Learn Syst. 2025;36(4):6995–7009. 10.1109/TNNLS.2024.3392355. 38748522

[29] Van Calster B, McLernon DJ, van Smeden M, Wynants L, Steyerberg EW. Topic Group ‘Evaluating diagnostic tests and prediction models’ of the STRATOS initiative. Calibration: the Achilles heel of predictive analytics. BMC Med. 2019;17(1):230. 10.1186/s12916-019-1466-7. Published 2019 Dec 16. PMC6912996 31842878

[30] Collins GS, Moons KGM, Dhiman P, et al. TRIPOD + AI statement: updated guidance for reporting clinical prediction models that use regression or machine learning methods. BMJ. 2024;385:e078378. 10.1136/bmj-2023-078378. Published 2024 Apr 16. 38626948 PMC11019967

[31] Mou W, Shan W, Yu S, et al. Interpretable machine learning model for predicting recurrence in patients with diabetic foot ulcers. BMJ Open Diabetes Res Care. 2025;13(6):e005242. 10.1136/bmjdrc-2025-005242. Published 2025 Nov 12. 41232937 PMC12612723

[32] Hoenigl M, Abramovitz D, Flores Ortega RE, Martin NK, Reau N. Sustained Impact of the Coronavirus Disease 2019 Pandemic on Hepatitis C Virus Treatment Initiations in the United States. Clin Infect Dis. 2022;75(1):e955–61. 10.1093/cid/ciac175. 35234860 PMC9129135

[33] Zibbell JE, Asher AK, Patel RC, et al. Increases in Acute Hepatitis C Virus Infection Related to a Growing Opioid Epidemic and Associated Injection Drug Use, United States, 2004 to 2014. Am J Public Health. 2018;108(2):175–81. 10.2105/AJPH.2017.304132. 29267061 PMC5846578

[34] Schaefer M, Capuron L, Friebe A, et al. Hepatitis C infection, antiviral treatment and mental health: a European expert consensus statement. J Hepatol. 2012;57(6):1379–90. 10.1016/j.jhep.2012.07.037. 22878466

[35] Cacoub P, Comarmond C, Domont F, Savey L, Desbois AC, Saadoun D. Extrahepatic manifestations of chronic hepatitis C virus infection. Ther Adv Infect Dis. 2016;3(1):3–14. 10.1177/2049936115585942. 26862398 PMC4735500

[36] Bassendine MF, Sheridan DA, Felmlee DJ, Bridge SH, Toms GL, Neely RD. HCV and the hepatic lipid pathway as a potential treatment target. J Hepatol. 2011;55(6):1428–40. 10.1016/j.jhep.2011.06.004. 21718665

[37] Mora Vallellano J, Cárdenas Lafuente F, Fernández Espínola S, et al. Closing the care gap – Systematic identification of overlooked patients with hepatitis C. Rev Esp Enferm Dig. 2025;117(11):705–6. 10.17235/reed.2025.11405/2025. 40613551

