# Supplementary material for: Development and validation of a machine learning model for evidence of prior or current HCV infection: a demographic screening approach for the US population
Source: BMC Public Health. 2026 Jul 1;26:2620. doi: 10.1186/s12889-026-28311-2 (PMC13587525; doi:10.1186/s12889-026-28311-2)
Supplement: Supplementary file 1 [file 12889_2026_28311_MOESM1_ESM.docx]

**Supplementary Table 1. Model details.**

| Model | Description | Implementation | Class Imbalance Handling |
| --- | --- | --- | --- |
| LR | Standard logistic regression; models additive linear associations on the log-odds scale. | stats::glm(family = binomial()); predictors: birth year, sex, race/ethnicity, US nativity, income-to-poverty ratio. | None. |
| WLR | Identical to LR but fit under the NHANES complex survey design. | survey::svyglm(family = quasibinomial()); same predictors with NHANES MEC examination weights, strata, and PSU. | NHANES MEC examination weights applied. |
| GAM | Extends LR by allowing a smooth nonlinear effect for birth year; remaining predictors are additive. | mgcv::gam(); s(birth_year, k = 5) + linear terms; REML estimation. | None. |
| RF | Ensemble of decision trees capturing nonlinear effects and interactions automatically. | ranger probability forest; 1,000 trees, mtry = 2, min. node size = 10. | Training set downsampled to equal case/non-case counts. |
| XGB | Sequentially boosted shallow trees; captures nonlinear effects and interactions. | xgboost::xgb.train; eta = 0.05, max depth = 3; maximum 300 rounds; boosting rounds selected by 5-fold cross-validation within the training cohort with early stopping after 30 rounds; validation cohort not used for tuning. | scale_pos_weight set to ratio of negatives to positives. |
| GLMNET | Penalized logistic regression; shrinks weak predictors to reduce overfitting. | glmnet::cv.glmnet(family = binomial, alpha = 0.5); lambda selected by 5-fold CV maximizing AUC. | None. |

**Supplementary Table 2. Baseline characteristics of current vs previous HCV infection among HCV antibody-positive participants with RNA results.**

| Variable | HCV antibody-positive with RNA result  (n = 888) | Current HCV infection  (n = 580) | Previous HCV infection  (n = 308) | p value |
| --- | --- | --- | --- | --- |
| **Male sex** | 62.50 | 66.03 | 55.84 | 0.003 |
| **Birth year** | 1958 (1952-1965) | 1957 (1951-1964) | 1960 (1955-1965) | <0.001 |
| **1911-1930** | 1.80 | 2.24 | 0.97 |  |
| **1931-1950** | 18.58 | 21.38 | 13.31 |  |
| **1951-1970** | 67.68 | 65.17 | 72.40 |  |
| **1971-1990** | 10.81 | 10.52 | 11.36 |  |
| **1991-2010** | 1.13 | 0.69 | 1.95 |  |
| **Race/ethnicity** |  |  |  | <0.001 |
| **Non-Hispanic White** | 42.34 | 38.97 | 48.70 |  |
| **Non-Hispanic Black** | 35.02 | 39.83 | 25.97 |  |
| **Hispanic** | 16.55 | 16.72 | 16.23 |  |
| **Asian or other** | 6.08 | 4.48 | 9.09 |  |
| **Birth place: USA-born** | 92.45 | 93.10 | 91.23 | 0.315 |
| **Education (n = 883)** |  |  |  | 0.013 |
| **Less than college** | 93.09 | 94.63 | 90.20 |  |
| **College or above** | 6.91 | 5.37 | 9.80 |  |
| **Health insurance**  **(n = 676)** |  |  |  | <0.001 |
| **Yes** | 75.44 | 69.73 | 83.88 |  |
| **No** | 24.56 | 30.27 | 16.12 |  |
| **Marital status (n = 880)** |  |  |  | 0.871 |
| **Married or living with partner** | 46.93 | 47.13 | 46.56 |  |
| **Widowed, divorced, separated, or never married** | 53.07 | 52.87 | 53.44 |  |
| **Alcohol use in past 12 months (n = 535)** |  |  |  | 0.218 |
| **>1/day** | 82.24 | 83.66 | 79.31 |  |
| **<=1/day** | 17.76 | 16.34 | 20.69 |  |
| **Smoking (n = 638)** |  |  |  | 0.004 |
| **Every day or some days** | 74.76 | 78.05 | 67.35 |  |
| **Never** | 25.24 | 21.95 | 32.65 |  |
| **Depression (n = 620)** |  |  |  | 0.873 |
| **Probable case** | 21.29 | 21.51 | 20.97 |  |
| **Not a probable case** | 78.71 | 78.49 | 79.03 |  |
| **Diabetes (n = 870)** | 15.86 | 13.49 | 20.40 | 0.008 |
| **CAD (n = 881)** | 6.47 | 5.72 | 7.89 | 0.212 |
| **Income to poverty ratio**  **(n = 888)** | 1.3  (0.8-2.4) | 1.2  (0.8-2.2) | 1.4  (0.9-2.9) | 0.010 |
| **BMI, kg/m2 (n = 863)** | 27.2  (23.5-31.2) | 26.6  (23.4-30.6) | 28.0  (23.8-32.2) | 0.026 |
| **HbA1c, % (n = 885)** | 5.5  (5.2-5.9) | 5.4  (5.2-5.8) | 5.6  (5.3-6.0) | 0.001 |
| **Glucose, mg/dl (n = 431)** | 102.0  (93.1-113.0) | 101.5  (93.0-112.8) | 104.0  (94.6-113.0) | 0.219 |
| **HDL, mg/dl (n = 744)** | 50.0  (40.8-63.0) | 50.0  (40.0-65.0) | 49.0  (41.0-60.0) | 0.489 |
| **LDL, mg/dl (n = 413)** | 97.0  (80.0-126.0) | 95.0  (78.0-119.0) | 107.5  (83.2-138.8) | 0.001 |
| **TG, mg/dl (n = 431)** | 97.0  (69.0-144.0) | 94.0  (68.0-134.5) | 102.0  (71.0-165.0) | 0.018 |
| **ALT, U/L (n = 877)** | 32.0  (21.0-54.0) | 44.0  (29.0-72.0) | 19.0  (14.0-25.0) | <0.001 |
| **AST, U/L (n = 877)** | 33.0  (23.0-50.0) | 42.0  (30.8-64.2) | 22.0  (18.0-27.0) | <0.001 |
| **ALP, U/L (n = 877)** | 75.0  (61.0-94.0) | 75.0  (62.0-95.2) | 75.0  (60.0-94.0) | 0.484 |
| **GGT, U/L (n = 877)** | 39.0  (22.0-86.0) | 57.5  (29.8-119.0) | 24.0  (17.0-36.0) | <0.001 |
| **TBil, mg/dl (n = 781)** | 0.6  (0.4-0.8) | 0.7  (0.5-0.9) | 0.5  (0.4-0.7) | <0.001 |
| **Creatinine, mg/dl**  **(n = 877)** | 0.9  (0.7-1.0) | 0.9  (0.7-1.0) | 0.8  (0.7-1.0) | 0.167 |
| **Platelets, x10^9/L**  **(n = 885)** | 229.0  (179.0-277.0) | 224.0  (177.5-269.0) | 239.5  (187.2-296.0) | 0.005 |

Categorical variables are shown as percentages; continuous variables are shown as median (Q1-Q3). Variable-specific sample sizes are shown in the row labels because data were not available for all participants across all NHANES cycles. P values compare current HCV infection and past HCV infection participants. HCV, hepatitis C virus; CAD, coronary artery disease; BMI, body mass index; HbA1c, hemoglobin A1c; HDL, high-density lipoprotein; LDL, low-density lipoprotein; TG, triglycerides; ALT, alanine aminotransferase; AST, aspartate aminotransferase; ALP, alkaline phosphatase; GGT, gamma-glutamyl transferase; TBil, total bilirubin.

**Supplementary Table 3. Baseline characteristics of training and validation cohorts.**

| Variable | Training  (n = 48,434) | Validation  (n = 20,762) | p value |
| --- | --- | --- | --- |
| **Male sex** | 49.33 | 47.69 | <0.001 |
| **Birth year** | 1976 (1954-1992) | 1976 (1955-1990) | <0.001 |
| **1911-1930** | 4.13 | 5.59 |  |
| **1931-1950** | 17.15 | 14.54 |  |
| **1951-1970** | 23.23 | 25.21 |  |
| **1971-1990** | 28.68 | 33.67 |  |
| **1991-2010** | 26.81 | 21.00 |  |
| **Race/ethnicity** |  |  | <0.001 |
| **Non-Hispanic White** | 40.27 | 46.80 |  |
| **Non-Hispanic Black** | 23.18 | 20.84 |  |
| **Hispanic** | 27.20 | 25.75 |  |
| **Asian or other** | 9.35 | 6.61 |  |
| **Birth place: USA-born** | 81.71 | 82.71 | 0.002 |
| **Education (n = 54,663)** |  |  | <0.001 |
| **Less than college** | 80.46 | 77.76 |  |
| **College or above** | 19.54 | 22.24 |  |
| **Health insurance (n = 48,516)** |  |  | <0.001 |
| **Yes** | 82.19 | 85.71 |  |
| **No** | 17.81 | 14.29 |  |
| **Marital status (n = 51,622)** |  |  | <0.001 |
| **Married or living with partner** | 54.96 | 50.74 |  |
| **Widowed, divorced, separated, or never married** | 45.04 | 49.26 |  |
| **Alcohol use in past 12 months (n = 29,693)** |  |  | 0.477 |
| **>1/day** | 64.99 | 65.42 |  |
| **<=1/day** | 35.01 | 34.58 |  |
| **Smoking (n = 35,220)** |  |  | 0.010 |
| **Every day or some days** | 27.41 | 26.06 |  |
| **Never** | 72.59 | 73.94 |  |
| **Depression (n = 32,990)** |  |  | 0.104 |
| **Probable case** | 9.19 | 9.78 |  |
| **Not a probable case** | 90.81 | 90.22 |  |
| **Diabetes (n = 68,046)** | 8.65 | 7.59 | <0.001 |
| **CAD (n = 46,223)** | 4.48 | 4.24 | 0.251 |
| **Income to poverty ratio**  **(n = 69,196)** | 1.9  (1.0-3.8) | 2.3  (1.1-4.1) | <0.001 |
| **BMI, kg/m2 (n = 68,202)** | 26.0  (21.6-30.9) | 25.9  (21.6-30.6) | 0.018 |
| **HbA1c, % (n = 60,318)** | 5.4  (5.1-5.7) | 5.3  (5.1-5.6) | <0.001 |
| **Glucose, mg/dl (n = 29,644)** | 98.0  (91.0-107.0) | 97.0  (90.0-105.0) | <0.001 |
| **HDL, mg/dl (n = 55,039)** | 51.0  (43.0-61.0) | 52.0  (44.0-63.0) | <0.001 |
| **LDL, mg/dl (n = 29,569)** | 104.0  (83.0-129.0) | 104.0  (82.0-130.0) | 0.544 |
| **TG, mg/dl (n = 30,485)** | 95.0  (65.0-143.0) | 98.0  (68.0-147.0) | <0.001 |
| **ALT, U/L (n = 59,613)** | 19.0  (15.0-26.0) | 19.0  (14.0-26.0) | <0.001 |
| **AST, U/L (n = 59,568)** | 22.0  (19.0-27.0) | 22.0  (18.0-26.0) | <0.001 |
| **ALP, U/L (n = 59,700)** | 73.0  (58.0-93.0) | 77.0  (61.0-100.0) | <0.001 |
| **GGT, U/L (n = 59,699)** | 18.0  (13.0-28.0) | 18.0  (13.0-27.0) | <0.001 |
| **TBil, mg/dl (n = 53,233)** | 0.6  (0.4-0.8) | 0.5  (0.4-0.7) | <0.001 |
| **Creatinine, mg/dl (n = 59,704)** | 0.8  (0.7-1.0) | 0.8  (0.7-1.0) | <0.001 |
| **Platelets, x10^9/L (n = 69,106)** | 252.0  (213.0-298.0) | 271.0  (229.0-320.0) | <0.001 |

Categorical variables are shown as percentages; continuous variables are shown as median (Q1-Q3). Variable-specific sample sizes are shown in the row labels because data were not available for all participants across all NHANES cycles. P values compare training and validation cohorts. HCV, hepatitis C virus; CAD, coronary artery disease; BMI, body mass index; HbA1c, hemoglobin A1c; HDL, high-density lipoprotein; LDL, low-density lipoprotein; TG, triglycerides; ALT, alanine aminotransferase; AST, aspartate aminotransferase; ALP, alkaline phosphatase; GGT, gamma-glutamyl transferase; TBil, total bilirubin.

**Supplementary Table 4. Survey-weighted validation performance sensitivity analysis**

| **Model** | **Unweighted AUROC** | **Survey-weighted AUROC** | **Survey-weighted Brier score** | **Weighted prevalence** |
| --- | --- | --- | --- | --- |
| **LR** | 0.762 | 0.761 | 0.0152 | 0.016 |
| **WLR** | 0.757 | 0.760 | 0.0151 | 0.016 |
| **GAM** | 0.856 | 0.822 | 0.0147 | 0.016 |
| **RF** | 0.848 | 0.816 | 0.0149 | 0.016 |
| **XGB** | 0.859 | 0.830 | 0.0147 | 0.016 |

Values are validation-cohort estimates. Weighted AUROC, weighted Brier score, and weighted prevalence were calculated using pooled NHANES mobile examination center weights. Primary machine-learning performance estimates were unweighted; this table provides a sensitivity analysis for population weighting. AUROC, area under the receiver operating characteristic curve.

**Supplementary Table 5. Model performance for current HCV infection prediction**

| **Cohort** | **Model** | **Sensitivity** | **Specificity** | **PPV** | **NPV** | **AUROC** |
| --- | --- | --- | --- | --- | --- | --- |
| **Training** | LR | 0.616 | 0.804 | 0.0275 | 0.996 | 0.802 |
|  | GAM | 0.791 | 0.805 | 0.0353 | 0.998 | 0.879 |
|  | GLMNET | 0.536 | 0.803 | 0.0240 | 0.995 | 0.793 |
|  | RF^*^ | 0.906 | 0.806 | 0.0404 | 0.999 | 0.921 |
|  | WLR | 0.614 | 0.804 | 0.0274 | 0.996 | 0.796 |
|  | XGB | 0.852 | 0.806 | 0.0380 | 0.998 | 0.907 |
| **Validation** | LR | 0.588 | 0.803 | 0.0227 | 0.996 | 0.794 |
|  | GAM | 0.750 | 0.804 | 0.0290 | 0.998 | 0.863 |
|  | GLMNET | 0.538 | 0.803 | 0.0208 | 0.996 | 0.776 |
|  | RF | 0.713 | 0.804 | 0.0275 | 0.997 | 0.852 |
|  | WLR | 0.594 | 0.803 | 0.0230 | 0.996 | 0.791 |
|  | XGB^*^ | 0.794 | 0.805 | 0.0307 | 0.998 | 0.867 |

Model performance was evaluated in the training and validation cohorts. Sensitivity, specificity, positive predictive value (PPV), and negative predictive value (NPV) were calculated using the prespecified classification threshold for each model. Asterisks indicate the best-performing model in each cohort according to AUROC.

**Supplementary Table 6. Raw data underlying Figure 3: event rates and case capture by predicted-risk decile**

| **Cohort** | **Model** | **Risk decile** | **n** | **Observed cases** | **Observed event rate, %** | **Mean predicted event rate, %** | **Percent cases captured, %** |
| --- | --- | --- | --- | --- | --- | --- | --- |
| **Training** | LR | 1 | 4,844 | 4 | 0.08 | 0.16 | 0.58 |
|  | LR | 2 | 4,844 | 9 | 0.19 | 0.30 | 1.32 |
|  | LR | 3 | 4,844 | 10 | 0.21 | 0.43 | 1.46 |
|  | LR | 4 | 4,844 | 19 | 0.39 | 0.57 | 2.78 |
|  | LR | 5 | 4,843 | 32 | 0.66 | 0.74 | 4.68 |
|  | LR | 6 | 4,843 | 48 | 0.99 | 0.95 | 7.02 |
|  | LR | 7 | 4,843 | 61 | 1.26 | 1.25 | 8.92 |
|  | LR | 8 | 4,843 | 102 | 2.11 | 1.70 | 14.91 |
|  | LR | 9 | 4,843 | 160 | 3.30 | 2.58 | 23.39 |
|  | LR | 10 | 4,843 | 239 | 4.93 | 5.45 | 34.94 |
| **Training** | XGB | 1 | 4,844 | 0 | 0.00 | 0.00 | 0.00 |
|  | XGB | 2 | 4,844 | 0 | 0.00 | 0.01 | 0.00 |
|  | XGB | 3 | 4,844 | 0 | 0.00 | 0.04 | 0.00 |
|  | XGB | 4 | 4,844 | 3 | 0.06 | 0.09 | 0.44 |
|  | XGB | 5 | 4,843 | 6 | 0.12 | 0.18 | 0.88 |
|  | XGB | 6 | 4,843 | 24 | 0.50 | 0.33 | 3.51 |
|  | XGB | 7 | 4,843 | 26 | 0.54 | 0.67 | 3.80 |
|  | XGB | 8 | 4,843 | 77 | 1.59 | 1.32 | 11.26 |
|  | XGB | 9 | 4,843 | 124 | 2.56 | 2.63 | 18.13 |
|  | XGB | 10 | 4,843 | 424 | 8.75 | 8.86 | 61.99 |
| **Validation** | LR | 1 | 2,077 | 3 | 0.14 | 0.15 | 1.06 |
|  | LR | 2 | 2,077 | 1 | 0.05 | 0.27 | 0.35 |
|  | LR | 3 | 2,076 | 7 | 0.34 | 0.38 | 2.47 |
|  | LR | 4 | 2,076 | 4 | 0.19 | 0.51 | 1.41 |
|  | LR | 5 | 2,076 | 14 | 0.67 | 0.66 | 4.95 |
|  | LR | 6 | 2,076 | 32 | 1.54 | 0.86 | 11.31 |
|  | LR | 7 | 2,076 | 32 | 1.54 | 1.14 | 11.31 |
|  | LR | 8 | 2,076 | 41 | 1.97 | 1.56 | 14.49 |
|  | LR | 9 | 2,076 | 63 | 3.03 | 2.38 | 22.26 |
|  | LR | 10 | 2,076 | 86 | 4.14 | 5.44 | 30.39 |
| **Validation** | XGB | 1 | 2,077 | 1 | 0.05 | 0.00 | 0.35 |
|  | XGB | 2 | 2,077 | 2 | 0.10 | 0.02 | 0.71 |
|  | XGB | 3 | 2,076 | 5 | 0.24 | 0.05 | 1.77 |
|  | XGB | 4 | 2,076 | 3 | 0.14 | 0.10 | 1.06 |
|  | XGB | 5 | 2,076 | 3 | 0.14 | 0.18 | 1.06 |
|  | XGB | 6 | 2,076 | 10 | 0.48 | 0.32 | 3.53 |
|  | XGB | 7 | 2,076 | 15 | 0.72 | 0.65 | 5.30 |
|  | XGB | 8 | 2,076 | 32 | 1.54 | 1.28 | 11.31 |
|  | XGB | 9 | 2,076 | 50 | 2.41 | 2.35 | 17.67 |
|  | XGB | 10 | 2,076 | 162 | 7.80 | 7.85 | 57.24 |

Values are aggregate source data used to draw Figure 3. Deciles were defined within each cohort and model using Platt-calibrated predicted risks, with decile 1 representing the lowest predicted risk and decile 10 the highest predicted risk. Observed event rate is the percentage of participants within a decile who were HCV-marker positive. Mean predicted event rate is the mean calibrated predicted risk within a decile. Percent cases captured is the percentage of all HCV-marker-positive participants in the corresponding cohort/model panel who fell within that decile; case-capture percentages sum to 100% across deciles within each panel. LR, logistic regression; XGB, XGBoost.

**Supplementary Table 7. Birth-year-only sensitivity analysis**

| **Model** | **Validation n/events** | **AUROC (95% CI)** | **p vs full XGBoost** | **PR-AUC** | **Brier score** | **Sensitivity (95% CI)** | **Specificity (95% CI)** | **PPV (95% CI)** | **Top-decile prevalence** | **Top-decile case capture** |
| --- | --- | --- | --- | --- | --- | --- | --- | --- | --- | --- |
| **Full XGBoost (five variables)** | 20762/283 | 0.859 (0.839-0.879) | Reference | 0.092 | 0.013 | 74.9% (69.6-79.6) | 80.8% (80.2-81.3) | 5.1% (4.5-5.8) | 7.8% | 57.2% |
| **Full LR (five variables)** | 20762/283 | 0.762 (0.738-0.785) | <0.001 | 0.035 | 0.013 | 52.7% (46.8-58.4) | 80.4% (79.9-81.0) | 3.6% (3.1-4.2) | 4.1% | 30.4% |
| **Birth year only XGBoost** | 20762/283 | 0.790 (0.768-0.811) | <0.001 | 0.041 | 0.013 | 61.8% (56.1-67.3) | 79.6% (79.0-80.1) | 4.0% (3.5-4.6) | 4.4% | 32.5% |
| **Birth year only GAM** | 20762/283 | 0.790 (0.768-0.812) | <0.001 | 0.041 | 0.013 | 59.4% (53.6-64.9) | 79.9% (79.3-80.4) | 3.9% (3.4-4.5) | 4.4% | 32.5% |
| **Birth year only LR** | 20762/283 | 0.687 (0.669-0.705) | <0.001 | 0.020 | 0.013 | 18.7% (14.6-23.7) | 79.4% (78.8-79.9) | 1.2% (0.9-1.6) | 0.8% | 6.4% |
| **Birth cohort category only LR** | 20762/283 | 0.772 (0.750-0.795) | <0.001 | 0.043 | 0.013 | 70.0% (64.4-75.0) | 76.1% (75.5-76.7) | 3.9% (3.4-4.5) | 3.9% | 70.0% |

Values are validation-cohort estimates. Birth-year-only models were trained using birth year as the sole predictor, except the birth-cohort category model, which used categorical birth-cohort groups. For the birth-year-only XGBoost model, boosting rounds were selected by 5-fold cross-validation within the training cohort only, without using the validation cohort for early stopping. Threshold-dependent metrics used each model's validation 80th-percentile predicted-risk cutoff for fixed-capacity comparison. The birth-cohort category model creates tied predicted risks, so top-decile case-capture values for that row should be interpreted cautiously. AUROC, area under the receiver operating characteristic curve; PR-AUC, area under the precision-recall curve; PPV, positive predictive value.

**Supplementary Table 8. XGBoost validation performance by subgroup**

**Panel A. Discrimination and threshold-dependent performance**

| **Subgroup variable** | **Subgroup** | **n/events** | **Observed prevalence** | **AUROC (95% CI)** | **Sensitivity (95% CI)** | **False-negative rate (95% CI)** | **Specificity (95% CI)** | **PPV (95% CI)** | **High-risk classified** |
| --- | --- | --- | --- | --- | --- | --- | --- | --- | --- |
| **Sex** | Female | 10860/113 | 1.0% | 0.829 (0.791-0.868) | 61.9% (52.7-70.4) | 38.1% (29.6-47.3) | 86.4% (85.8-87.1) | 4.6% (3.6-5.8) | 14.1% |
|  | Male | 9902/170 | 1.7% | 0.874 (0.853-0.896) | 83.5% (77.2-88.4) | 16.5% (11.6-22.8) | 74.5% (73.6-75.3) | 5.4% (4.6-6.3) | 26.5% |
| **Race/ethnicity** | Asian/Other | 1373/24 | 1.7% | 0.797 (0.707-0.887) | 54.2% (35.1-72.1) | 45.8% (27.9-64.9) | 84.4% (82.4-86.3) | 5.8% (3.4-9.7) | 16.2% |
|  | Black | 4326/85 | 2.0% | 0.888 (0.862-0.914) | 92.9% (85.4-96.7) | 7.1% (3.3-14.6) | 70.5% (69.1-71.9) | 5.9% (4.8-7.3) | 30.7% |
|  | Hispanic | 5347/52 | 1.0% | 0.882 (0.837-0.927) | 61.5% (48.0-73.5) | 38.5% (26.5-52.0) | 90.6% (89.8-91.4) | 6.1% (4.3-8.4) | 9.9% |
|  | White | 9716/122 | 1.3% | 0.835 (0.801-0.868) | 72.1% (63.6-79.3) | 27.9% (20.7-36.4) | 79.3% (78.5-80.1) | 4.2% (3.5-5.2) | 21.3% |
| **Birthplace** | Non-US-born | 3590/21 | 0.6% | 0.786 (0.705-0.867) | 23.8% (10.6-45.1) | 76.2% (54.9-89.4) | 92.9% (92.0-93.7) | 1.9% (0.8-4.5) | 7.2% |
|  | US-born | 17172/262 | 1.5% | 0.860 (0.839-0.880) | 79.0% (73.7-83.5) | 21.0% (16.5-26.3) | 78.2% (77.6-78.8) | 5.3% (4.7-6.1) | 22.7% |
| **Birth year** | 1911-1930 | 1128/6 | 0.5% | 0.912 (0.855-0.969) | 50.0% (18.8-81.2) | 50.0% (18.8-81.2) | 89.9% (88.0-91.6) | 2.6% (0.9-7.3) | 10.3% |
|  | 1931-1950 | 2934/37 | 1.3% | 0.697 (0.614-0.781) | 51.4% (35.9-66.6) | 48.6% (33.4-64.1) | 69.9% (68.2-71.5) | 2.1% (1.4-3.3) | 30.4% |
|  | 1951-1970 | 5088/198 | 3.9% | 0.769 (0.739-0.799) | 91.4% (86.7-94.6) | 8.6% (5.4-13.3) | 43.1% (41.7-44.5) | 6.1% (5.3-7.0) | 58.3% |
|  | 1971-1990 | 6795/36 | 0.5% | 0.774 (0.680-0.868) | 25.0% (13.8-41.1) | 75.0% (58.9-86.2) | 97.5% (97.1-97.8) | 5.0% (2.7-9.2) | 2.6% |
|  | 1991-2010 | 4238/6 | 0.1% | 0.766 (0.553-0.980) | 0.0% (0.0-39.0) | 100.0% (61.0-100.0) | 100.0% (99.9-100.0) | NA | 0.0% |
| **Income to poverty ratio** | <=1.0 | 4363/91 | 2.1% | 0.870 (0.836-0.904) | 80.2% (70.9-87.1) | 19.8% (12.9-29.1) | 79.8% (78.6-81.0) | 7.8% (6.3-9.7) | 21.4% |
|  | >1.0-2.0 | 5055/95 | 1.9% | 0.852 (0.821-0.882) | 77.9% (68.6-85.1) | 22.1% (14.9-31.4) | 77.3% (76.1-78.4) | 6.2% (4.9-7.7) | 23.7% |
|  | >2.0-4.0 | 5844/64 | 1.1% | 0.829 (0.784-0.873) | 64.1% (51.8-74.7) | 35.9% (25.3-48.2) | 80.6% (79.5-81.6) | 3.5% (2.6-4.7) | 19.9% |
|  | >4.0 | 5500/33 | 0.6% | 0.840 (0.761-0.919) | 72.7% (55.8-84.9) | 27.3% (15.1-44.2) | 84.8% (83.9-85.8) | 2.8% (1.9-4.2) | 15.5% |
|  |  |  |  |  |  |  |  |  |  |
|  |  |  |  |  |  |  |  |  |  |

Panel A reports validation-cohort performance at the validation cohort 80th-percentile XGBoost raw-score threshold used for the fixed-capacity comparison in Table 3. PPV, positive predictive value; AUROC, area under the receiver operating characteristic curve.

**Panel B. Calibration by subgroup**

| **Subgroup variable** | **Subgroup** | **n/events** | **Observed prevalence** | **Mean predicted risk** | **Brier score** | **Calibration intercept (95% CI)** | **Calibration slope (95% CI)** |
| --- | --- | --- | --- | --- | --- | --- | --- |
| **Sex** | Female | 10860/113 | 1.0% | 0.9% | 0.010 | 0.139 (-0.050-0.328) | 0.750 (0.617-0.882) |
|  | Male | 9902/170 | 1.7% | 1.7% | 0.016 | 0.022 (-0.135-0.179) | 0.939 (0.816-1.063) |
| **Race/ethnicity** | Asian/Other | 1373/24 | 1.7% | 1.1% | 0.017 | 0.520 (0.098-0.943) | 0.523 (0.302-0.743) |
|  | Black | 4326/85 | 2.0% | 2.0% | 0.018 | 0.005 (-0.217-0.227) | 1.042 (0.835-1.249) |
|  | Hispanic | 5347/52 | 1.0% | 0.6% | 0.009 | 0.460 (0.180-0.739) | 1.043 (0.806-1.279) |
|  | White | 9716/122 | 1.3% | 1.4% | 0.012 | -0.088 (-0.271-0.095) | 0.824 (0.692-0.956) |
| **Birthplace** | Non-US-born | 3590/21 | 0.6% | 0.5% | 0.006 | 0.068 (-0.363-0.499) | 0.820 (0.383-1.258) |
|  | US-born | 17172/262 | 1.5% | 1.4% | 0.014 | 0.068 (-0.058-0.194) | 0.844 (0.752-0.937) |
| **Birth year** | 1911-1930 | 1128/6 | 0.5% | 0.7% | 0.005 | -0.305 (-1.111-0.500) | 1.762 (0.774-2.750) |
|  | 1931-1950 | 2934/37 | 1.3% | 1.7% | 0.013 | -0.315 (-0.642-0.013) | 0.653 (0.350-0.955) |
|  | 1951-1970 | 5088/198 | 3.9% | 3.7% | 0.036 | 0.060 (-0.085-0.206) | 0.952 (0.801-1.102) |
|  | 1971-1990 | 6795/36 | 0.5% | 0.3% | 0.005 | 0.682 (0.351-1.013) | 0.885 (0.642-1.127) |
|  | 1991-2010 | 4238/6 | 0.1% | 0.0% | 0.001 | 1.315 (0.513-2.116) | 0.922 (0.162-1.681) |
| **Income to poverty ratio** | <=1.0 | 4363/91 | 2.1% | 2.1% | 0.019 | -0.015 (-0.233-0.202) | 0.780 (0.634-0.925) |
|  | >1.0-2.0 | 5055/95 | 1.9% | 1.6% | 0.018 | 0.197 (-0.012-0.406) | 0.823 (0.662-0.984) |
|  | >2.0-4.0 | 5844/64 | 1.1% | 1.0% | 0.011 | 0.116 (-0.134-0.366) | 0.806 (0.600-1.013) |
|  | >4.0 | 5500/33 | 0.6% | 0.7% | 0.006 | -0.115 (-0.459-0.229) | 1.069 (0.653-1.485) |

Panel B reports calibration using Platt-calibrated XGBoost predicted risks. Calibration estimates are descriptive and should be interpreted cautiously in sparse subgroups with few HCV-marker-positive participants.

**Supplementary Table 9. XGBoost validation performance across calibrated-risk thresholds**

| **Risk threshold** | **High-risk group** | **Sensitivity (95% CI)** | **Specificity (95% CI)** | **PPV (95% CI)** | **NPV (95% CI)** | **Case capture** | **NNS** | **XGB net benefit** | **XGB-LR gain per 1,000** |
| --- | --- | --- | --- | --- | --- | --- | --- | --- | --- |
| 1.50% | 4537 (21.9%) | 77.7% (72.5%-82.2%) | 78.9% (78.4%-79.5%) | 4.8% (4.3%-5.5%) | 99.6% (99.5%-99.7%) | 77.7% | 20.6 | 0.0074 | 3.0 |
| 2.00% | 3568 (17.2%) | 71.4% (65.9%-76.3%) | 83.6% (83.0%-84.1%) | 5.7% (4.9%-6.5%) | 99.5% (99.4%-99.6%) | 71.4% | 17.7 | 0.0064 | 3.3 |
| 2.50% | 2762 (13.3%) | 64.3% (58.6%-69.7%) | 87.4% (86.9%-87.8%) | 6.6% (5.7%-7.6%) | 99.4% (99.3%-99.5%) | 64.3% | 15.2 | 0.0056 | 3.4 |
| 3.00% | 2354 (11.3%) | 60.1% (54.3%-65.6%) | 89.3% (88.9%-89.8%) | 7.2% (6.2%-8.3%) | 99.4% (99.3%-99.5%) | 60.1% | 13.8 | 0.0049 | 3.8 |
| 3.50% | 2073 (10.0%) | 57.2% (51.4%-62.9%) | 90.7% (90.3%-91.1%) | 7.8% (6.7%-9.0%) | 99.4% (99.2%-99.5%) | 57.2% | 12.8 | 0.0045 | 4.0 |
| 3.75% | 1984 (9.6%) | 56.2% (50.4%-61.8%) | 91.1% (90.7%-91.5%) | 8.0% (6.9%-9.3%) | 99.3% (99.2%-99.4%) | 56.2% | 12.5 | 0.0042 | 4.3 |
| 4.00% | 1894 (9.1%) | 53.7% (47.9%-59.4%) | 91.5% (91.1%-91.9%) | 8.0% (6.9%-9.3%) | 99.3% (99.2%-99.4%) | 53.7% | 12.5 | 0.0038 | 3.9 |
| 4.50% | 1672 (8.1%) | 50.2% (44.4%-56.0%) | 92.5% (92.2%-92.9%) | 8.5% (7.2%-9.9%) | 99.3% (99.1%-99.4%) | 50.2% | 11.8 | 0.0034 | 3.8 |
| 5.00% | 1417 (6.8%) | 43.5% (37.8%-49.3%) | 93.7% (93.3%-94.0%) | 8.7% (7.3%-10.3%) | 99.2% (99.0%-99.3%) | 43.5% | 11.5 | 0.0026 | 3.0 |

Thresholds are Platt-calibrated XGBoost predicted-risk thresholds. The 80th-percentile cutoff used in Table 3 was retained as an illustrative fixed-capacity comparison and was not intended to define a deployable clinical threshold. Net-benefit gain is XGBoost minus standard logistic regression per 1,000 participants. PPV, positive predictive value; NPV, negative predictive value; NNS, number needed to screen.

**Supplementary Figure 1. Study flow.**

**Supplementary Figure 2. HCV infection risk and decile web calculator.**

**Supplementary Figure 3. Weighted HCV prevalence across NHANES cycle year by sex and by race/ethnicity.**

p <0.001

Prevalence (%)

| NHANES Cycle Year | Male | Female |
| --- | --- | --- |
| 1999-2000 | 1.81919% | 0.74199% |
| 2001-2002 | 2.39026% | 1.49754% |
| 2003-2004 | 1.77167% | 1.31711% |
| 2005-2006 | 1.70646% | 0.951348% |
| 2007-2008 | 1.78842% | 1.09692% |
| 2009-2010 | 1.89411% | 0.751625% |
| 2011-2012 | 1.84858% | 1.11364% |
| 2013-2014 | 1.6461% | 0.956699% |
| 2017-2020 | 2.08909% | 1.34625% |
| 2021-2023 | 1.55693% | 1.21571% |

p <0.001

Prevalence (%)

| NHANES Cycle Year | Hispanic | Non-Hispanic White | Non-Hispanic Black | Asian/Other |
| --- | --- | --- | --- | --- |
| 1999-2000 | 1.05894% | 1.09109% | 3.06256% | 0.619736% |
| 2001-2002 | 1.26524% | 1.91138% | 3.32464% | 1.00548% |
| 2003-2004 | 1.41168% | 1.47012% | 2.24305% | 1.20312% |
| 2005-2006 | 1.13354% | 1.15885% | 2.34462% | 1.56372% |
| 2007-2008 | 0.919004% | 1.32046% | 2.84581% | 0.970927% |
| 2009-2010 | 1.05265% | 1.18688% | 2.49182% | 1.03626% |
| 2011-2012 | 0.826149% | 1.68281% | 1.8087% | 0.445564% |
| 2013-2014 | 1.01311% | 1.22474% | 2.08131% | 1.29554% |
| 2017-2020 | 0.818094% | 1.96919% | 1.93773% | 1.30523% |
| 2021-2023 | 0.861866% | 1.23799% | 2.24728% | 2.09448% |

**Supplementary Figure 4. Calibration plots**

**Supplementary Figure 5. ROC curve comparison across models for current infection.**

**Supplementary Figure 6. Decision-curve analysis**
